# Is Auricular Stimulation Actually Useful in Reducing Preoperative Anxiety?

**DOI:** 10.3389/fpsyt.2022.854857

**Published:** 2022-04-15

**Authors:** Jing Dong, Yan-Chun Liao, Xiang Chen, Xin Ye, Yi-Feng Ren

**Affiliations:** ^1^Hospital of Chengdu University of Traditional Chinese Medicine, Chengdu, China; ^2^Department of Cardiology, Tianjin Baodi Hospital, Tianjin, China

**Keywords:** anxiety, auricular stimulation, meta-analysis, minimal clinically important difference, preoperative

## Introduction

The majority of patients commonly suffer from varying levels of exposure to physical and psychological stress during the perioperative period (before and after surgery). Approximately 80% of patients admitted for surgery suffered anxiety in the preoperative phase due to fear of pain, complications, or death, etc (1). It could cause further associated physical changes, which in turn influences postoperative recovery and increases complications of surgery and anesthesia (1, 2). Therefore, statistics-based valid assessment and effective clinical intervention for preoperative anxiety have always been regarded as significant issues for anesthesiologists and psychologists (3). Conventionally, therapy of preoperative anxiety has relied on various psychosocial and pharmaceutical interventions, such as anxiolytics, antidepressants, or monoamine oxidase inhibitors (4). Recently, a great deal of emphasis has been placed on non-pharmacological methods for preoperative anxiety, which are thought to benefit from multimodal analgesia and have fewer adverse effects (5). Among them, auricular stimulation (AS) as a method of complementary medicine, which is widely thought to contribute to anxiety therapy by stimulating the central nervous system through the auricle of the ear (6). Although several clinical trials have found the safety and superiority of AS, the heterogeneity with regard to surgical procedures, control conditions, and effect size makes it difficult to draw any definitive clinical recommendations.

## Comparison With Other Studies

A current review by Usichenko et al. represents an update on AS application’s effect of preoperative anxiety (7). The authors came to the conclusion that AS may be useful in the treatment of preoperative anxiety. However, some methodological issues captured our attention, including the unclear inclusion criteria, redundant effect sizes, and inappropriate combination of different effect sizes that might lead to biased results. We applaud the authors’ hard work, meanwhile updated and modified accordingly.

Specifically, there were some minor shortcomings that cannot be neglected in terms of methodology: (a) the flowchart did not follow the PRISMA guidelines. Specifically, the authors mentioned that five databases were searched in this study, but lack the identification data for each database. Besides, the results of each database filter were not recorded in the Supplementary Material; (b) the PICOS format (Participants, Intervention, Comparison, Outcomes, and Study design) was not sufficiently followed, and in particular to the main components of inclusion criteria (participants and comparison) remain vague. Conducting a systematic review with vague inclusion criteria may lead to problems concerning the validity and applicability of the systematic review;

(c) funnel plots were essential to determine the publication bias in the study, which were also not provided by the authors. We would like to suggest that funnel plots should be visually inspected, and Egger’s linear regression test should be performed to assess publication bias if at least five trials were identified; (d) the authors confused the concepts of sensitivity and subgroup analysis in contents of heterogeneity section, which resulted in increased reading difficulty. Considering the number of studies included, we suggest the use of a multivariate meta-regression model to detect the source of heterogeneity for the subgroups, rather than a single subgroup-analysis (8); (e) according to the Cochrane handbook, the study by Dellovo et al. (9) may not be appropriate for inclusion in this meta-analysis. The fundamental reason is that the authors included endpoint assessments post-operatively as effect sizes, which is inconsistent with the purpose of this review. Explicitly, redundant effect sizes were included.

Further, based on a comprehensive review of all included studies, we also found that some important characteristics of the trials were not mentioned in the study by Usichenko et al. (7). Specifically, the following information: (f) the Spielberger State-Trait Anxiety Inventory (STAI) consists of the self-reported state STAI (STAI-S) for immediate levels of anxiety and the trait STAI (STAI-T) for “general anxiety.” Therefore, the STAI type should have been discussed in detail by the authors; (g) another critical piece of information lacking in the report is the time of anxiety assessment. We do not think that a pooled analysis was appropriate because the effect of AS greatly varied within a short time span. The immediate benefit may be overshadowed by the decrease in effect with time. This may also be a source of heterogeneity, and may have affected the interpretation of the results.

**FIGURE 1:**
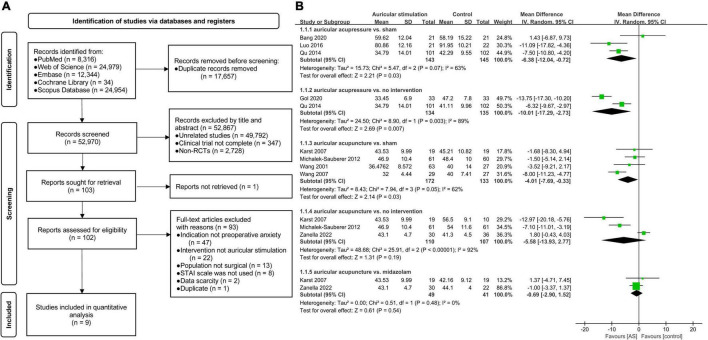
**(A)** Flowchart of study selection. **(B)** Forest plot of eleven trials comparing auricular stimulation with various control conditions. Anxiety was measured using State-Trait-Anxiety Inventory (STAI). IV, inverse variance; SD, standard deviation; CI, confidence interval; AS, auricular stimulation; vs., versus.

In summary, we conducted a reanalysis of the review by Usichenko et al. (7). The content accordingly was modified as follows:

(a) Comprehensive literature search was performed in MEDLINE (PubMed), EMBASE, Cochrane Central Register of Controlled Trials (CENTRAL), ISI Web of Science, Scopus Database until 8th March 2022 (see Figure 1A) followed the PRISMA guidelines.

(b) We referred to the inclusion criteria in the original literature but without language restriction. And the PICOS format was sufficiently followed: (i) Participants: included randomized clinical trials with patients from any population undergoing medical interventions under any type of anesthesia sedation, general anesthesia or locoregional anesthesia. (ii) Intervention: patients who had received AS or related interventions (e.g., auricular acupuncture, auricular acupressure, and auricular electroacupuncture) were included. (iii) Comparison: patients who had received any type of pharmacological or non-pharmacological control interventions were included. (iv) Outcomes: studies that reported anxiety scores were included. While studies not used STAI for anxiety scores were excluded. (v) Study design: the trials described full-text randomized controlled trials (RCTs) published in indexed or non-indexed journals. Ultimately, nine studies were included in the final analysis, the basic characteristics are presented in Supplementary Table 1.
(c) Publication bias was evaluated using a funnel plot analysis and Egger’s linear regression test. The results showed that there was no evidence of publication bias (*p* = 0.93) (Supplementary Figure 3). Moreover, we further assessed the risk of bias according to the revised Cochrane risk of bias tool version 2 (RoB 2) for RCTs. Additional details are available in Supplementary Figures 1, 2 and Supplementary Table 2).
(d) Subgroup analyses were performed based on the type of interventions (see Figure 1B and Supplementary Table 3), and meta-regression analysis was not performed due to the insufficient number of included studies.
(e) The study by Dellovo et al. (9) was excluded from our study.
(f) Detailed characteristics of studies were summarized in Supplementary Table 1, especially the STAI type were distinguished.
(g) The time of anxiety assessment was measured within 30 min after intervention in most studies, while some studies did not report the time-points of assessment (Supplementary Table 1).

## Discussion

It should be pointed out that the STAI scale was employed in most included trials in the review by Usichenko et al. (7), however, we are curious about why effect-sizes were summarized as standard mean difference (SMD) rather than weighted mean difference (WMD). WMD is a common statistic that calculates the absolute difference in mean values between two groups. It estimates the amount by which the experimental intervention affects the result on average when compared to the control group. When all studies’ outcomes are measured on the same scale, it can be used as a summary statistic in meta-analysis (10). Conversely, when the studies all assess the same outcome but quantify it in different ways, SMD is used as a summary statistic in meta-analysis. It should be noted that the method assumes that the differences in standard deviations among studies reflect differences in measurement scales and not real differences in variability among study populations (10). Undoubtedly, WMDs are able to provide a more clinically meaningful interpretation and degree of grasp for the pooled results and conclusions, according to the current general consensus (8). Specifically, to the best of our knowledge, a difference of 10% (8 points on the STAI) is the established minimal clinically important difference (MCID) for preoperative and postoperative anxiety (11). MCID is defined as the smallest change that is meaningful to patients and is considered the threshold needed to achieve treatment efficacy (12).

Therefore, we reran the primary outcome in this meta-analysis using the Inverse Variance Heterogeneity model with WMD (8), and also re-evaluated the results and conclusions according to the MCID in a clinical context. Finally, a clinically significant difference was detected in the reanalysis outcomes according to the pooled analysis of 9 included studies (4, 6, 13–19) (see Figure 1B). All included studies reported preoperative anxiety scores by STAI (total *N* = 711; AS group: *n* = 378; control group: *n* = 333). Differences in STAI between auricular acupressure and no intervention group were clinically significant difference [WMD = −10.01, 95% CI (−17.29 to −2.73), *p* = 0.007]. While the preoperative anxiety scores of auricular acupuncture group showed no statistical significance [WMD = −5.58, 95% CI (−13.93 to 2.77), *p* = 0.19], compared with no intervention group. When compared with sham group, new evidence suggested that the potential clinically superiority of auricular acupressure [WMD = −6.38, 95% CI (−12.04 to −0.72), *p* = 0.03] over auricular acupuncture [WMD = −4.01, 95% CI (−7.69 to −0.33), *p* = 0.03] in treatment of preoperative anxiety. However, neither of these outcome measurements reached MCID. Similarly, there was no statistically significant difference in STAI between the midazolam group and auricular acupuncture group [WMD = −0.69, 95% CI (−2.90 to 1.52), *p* = 0.54]. In summary, the reanalysis evidence further enhanced the clinical relevance of AS, while weakened the clinical effectiveness.

We respectfully appreciate that Usichenko et al. (7) provided us with an important meta-analysis which provides a guide for clinical decision-making. However, we conclude that the current review draws unreliability conclusions with the potential to exposure patients to the risk of unnecessary treatment. Hence, the correction of the stated faults may result in potential differences in the conclusions that can be drawn from the present meta-analysis.

## Author Contributions

JD and Y-CL conceived, designed, planned the study, and drafted the manuscript. Y-FR and XC supervised the study. Y-FR and XY critically revised the manuscript for important intellectual content. All authors have full access to the manuscript and take responsibility for the study design and approved the manuscript and agree with submission.

## Conflict of Interest

The authors declare that the research was conducted in the absence of any commercial or financial relationships that could be construed as a potential conflict of interest.

## Publisher’s Note

All claims expressed in this article are solely those of the authors and do not necessarily represent those of their affiliated organizations, or those of the publisher, the editors and the reviewers. Any product that may be evaluated in this article, or claim that may be made by its manufacturer, is not guaranteed or endorsed by the publisher.
