# Supplementary material for: Is Auricular Stimulation Actually Useful in Reducing Preoperative Anxiety?
Source: Front Psychiatry. 2022 Apr 15;13:854857. doi: 10.3389/fpsyt.2022.854857 (PMC9051329; doi:10.3389/fpsyt.2022.854857)
Supplement: Supplementary file 1 [file Data_Sheet_1.pdf]

Is auricular stimulation actually useful in reducing preoperative anxiety?

Supplementary Online Content

Table of Contents	Page
**eMethods 1:** PubMed Search Strategy Search strategy	**Page 2-3**
**eMethods 2:** Web of Science Search Strategy Search strategy	**Page 4**
**eMethods 3:** EMBASE Search Strategy Search strategy	**Page 5**
**eMethods 4:** Cochrane Library Search Strategy Search strategy	**Page 6**
**eMethods 5:** Scopus Database Search Strategy Search strategy	**Page 7**
**eTable 1:** Baseline characteristics of included studies	**Page 8-9**
**eTable 2:** Risk of Bias Assessment	**Page 10-13**
**eTable 3:** Predefined subgroup analyses for primary outcome	**Page 14**
**eFigure 1:** Risk of bias summary	**Page 15**
**eFigure 2:** Risk of bias graph	**Page 16**
**eFigure 3:** Funnel Plot	**Page 17**

eMethods 1: PubMed Search Strategy Search strategy

PubMed (8,316)

N	**Search item [title/ abstract]**
1	Randomized controlled trial
2	Controlled clinical trial
3	Randomized
4	Randomly
5	Trial
6	OR # 1-6
7	Anxiety
8	Fear
9	Preoperative
10	Surgical
11	Intervention
12	Anesthesia
13	OR # 7-12
14	Auricular acupuncture
15	Auricular
16	Ear
17	Acupressure
18	Electro-acupuncture
19	Stimulation
20	OR # 14-18

#1: ((((Randomized controlled trial[Title/Abstract]) OR (Controlled clinical trial[Title/Abstract])) OR (Randomized[Title/Abstract])) OR (Randomly[Title/Abstract])) OR (Trial[Title/Abstract]) [**1,288,816**]

#2: (((((Anxiety[Title/Abstract]) OR (Fear[Title/Abstract])) OR (Preoperative[Title/Abstract])) OR (Surgical[Title/Abstract])) OR (Intervention[Title/Abstract])) OR (Anesthesia[Title/Abstract]) [**2,282,257**]

#3: (((((Auricular acupuncture[Title/Abstract]) OR (Auricular[Title/Abstract])) OR (Ear[Title/Abstract])) OR (Acupressure[Title/Abstract])) OR (Electro-acupuncture[Title/Abstract])) OR (Stimulation[Title/Abstract]) **[707,405]**

#4: ((((((Randomized controlled trial[Title/Abstract]) OR (Controlled clinical trial[Title/Abstract])) OR (Randomized[Title/Abstract])) OR (Randomly[Title/Abstract])) OR (Trial[Title/Abstract])) AND ((((((Anxiety[Title/Abstract]) OR (Fear[Title/Abstract])) OR (Preoperative[Title/Abstract])) OR (Surgical[Title/Abstract])) OR (Intervention[Title/Abstract])) OR (Anesthesia[Title/Abstract]))) AND ((((((Auricular acupuncture[Title/Abstract]) OR (Auricular[Title/Abstract])) OR (Ear[Title/Abstract])) OR (Acupressure[Title/Abstract])) OR (Electro-acupuncture[Title/Abstract])) OR (Stimulation[Title/Abstract])) **[8,316]**

History and Search Details Download Delete

Search	Actions	Details	Query	Results	Time
#4	•••	>	Search: **((((((Randomized controlled trial[Title/Abstract]) OR (Controlled clinical trial[Title/Abstract])) OR (Randomized[Title/Abstract])) OR (Randomly[Title/Abstract])) OR (Trial[Title/Abstract])) AND ((((((Anxiety[Title/Abstract]) OR (Fear[Title/Abstract])) OR (Preoperative[Title/Abstract])) OR (Surgical[Title/Abstract])) OR (Intervention[Title/Abstract])) OR (Anesthesia[Title/Abstract]))) AND ((((((Auricular acupuncture[Title/Abstract]) OR (Auricular[Title/Abstract])) OR (Ear[Title/Abstract])) OR (Acupressure[Title/Abstract])) OR (Electro-acupuncture[Title/Abstract])) OR (Stimulation[Title/Abstract]))** Sort by: **Most Recent**	8,316	17:59:59
#3	•••	>	Search: **(((((Auricular acupuncture[Title/Abstract]) OR (Auricular[Title/Abstract])) OR (Ear[Title/Abstract])) OR (Acupressure[Title/Abstract])) OR (Electro-acupuncture[Title/Abstract])) OR (Stimulation[Title/Abstract])** Sort by: **Most Recent**	707,405	17:56:43
#2	•••	>	Search: **(((((Anxiety[Title/Abstract]) OR (Fear[Title/Abstract])) OR (Preoperative[Title/Abstract])) OR (Surgical[Title/Abstract])) OR (Intervention[Title/Abstract])) OR (Anesthesia[Title/Abstract])** Sort by: **Most Recent**	2,282,257	17:55:36
#1	•••	>	Search: **((((Randomized controlled trial[Title/Abstract]) OR (Controlled clinical trial[Title/Abstract])) OR (Randomized[Title/Abstract])) OR (Randomly[Title/Abstract])) OR (Trial[Title/Abstract])** Sort by: **Most Recent**	1,288,816	17:54:32

eMethods 2: Web of Science Search Strategy Search strategy

Web of Science (24,979)

#1: TOPIC: Randomized controlled trial (Topic) or Controlled clinical trial (Topic) or Randomized (Topic) or Randomly (Topic) or Trial (Topic) [**3,620,490**]

#2: TOPIC: Anxiety (Topic) or Fear (Topic) or Preoperative (Topic) or Surgical (Topic) or Intervention (Topic) or Anesthesia (Topic) [**5,201,551**]

#3: TOPIC: Auricular acupuncture (Topic) or Auricular (Topic) or Ear (Topic) or Acupressure (Topic) or Electro-acupuncture (Topic) or Stimulation (Topic) [**1,765,523**]

#4: #1 AND #2 AND #3 [**24,979**]

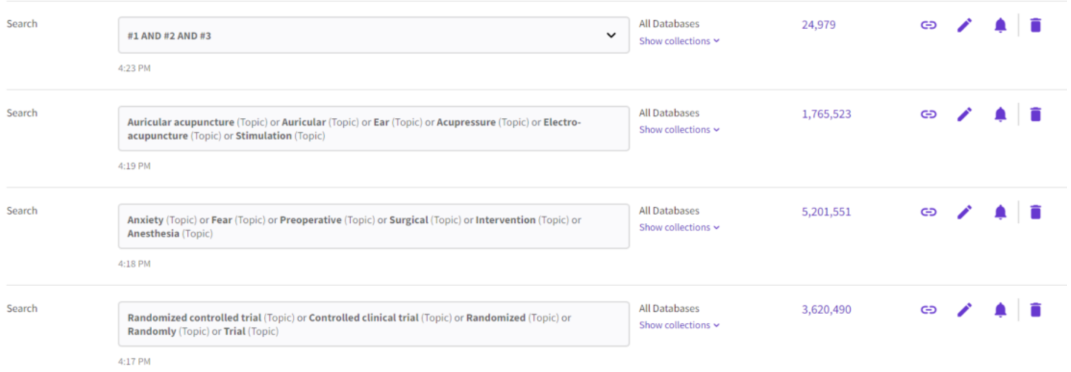

Search	#1 AND #2 AND #3<br>4:23 PM	All Databases<br>Show collections	24,979
Search	Auricular acupuncture (Topic) or Auricular (Topic) or Ear (Topic) or Acupressure (Topic) or Electro-acupuncture (Topic) or Stimulation (Topic)<br>4:19 PM	All Databases<br>Show collections	1,765,523
Search	Anxiety (Topic) or Fear (Topic) or Preoperative (Topic) or Surgical (Topic) or Intervention (Topic) or Anesthesia (Topic)<br>4:18 PM	All Databases<br>Show collections	5,201,551
Search	Randomized controlled trial (Topic) or Controlled clinical trial (Topic) or Randomized (Topic) or Randomly (Topic) or Trial (Topic)<br>4:17 PM	All Databases<br>Show collections	3,620,490

eMethods 3: EMBASE Search Strategy Search strategy

Embase (12,344)

#1: 'randomized controlled trial':ab,ti OR 'controlled clinical trial':ab,ti OR randomized:ab,ti OR randomly:ab,ti OR trial:ab,ti [**1,829,226**]

#2: anxiety:ab,ti OR fear:ab,ti OR preoperative:ab,ti OR surgical:ab,ti OR intervention:ab,ti OR anesthesia:ab,ti [**3,100,487**]

#3: anxiety:ab,ti OR fear:ab,ti OR preoperative:ab,ti OR surgical:ab,ti OR intervention:ab,ti OR anesthesia:ab,ti [**885,343**]

#4: #1 AND #2 AND #3 [**12,344**]

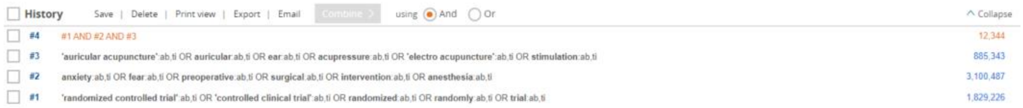
History Save | Delete | Print view | Export | Email Combine using And Or Collapse

#4	#1 AND #2 AND #3	12,344
#3	'auricular acupuncture':ab,ti OR auricular:ab,ti OR ear:ab,ti OR acupressure:ab,ti OR 'electro acupuncture':ab,ti OR stimulation:ab,ti	885,343
#2	anxiety:ab,ti OR fear:ab,ti OR preoperative:ab,ti OR surgical:ab,ti OR intervention:ab,ti OR anesthesia:ab,ti	3,100,487
#1	'randomized controlled trial':ab,ti OR 'controlled clinical trial':ab,ti OR randomized:ab,ti OR randomly:ab,ti OR trial:ab,ti	1,829,226

eMethods 4: Cochrane Library Search Strategy Search strategy

Cochrane Library (34)

#1: (Randomized controlled trial):ti,ab,kw OR (Controlled clinical trial):ti,ab,kw OR (Randomized):ti,ab,kw OR (Randomly):ti,ab,kw OR (Trial):ti,ab,kw [**1020**]

#2: (Anxiety):ti,ab,kw OR (Fear):ti,ab,kw OR (Preoperative):ti,ab,kw OR (Surgical):ti,ab,kw OR (Intervention):ti,ab,kw OR (Anesthesia):ti,ab,kw [**591290**]

#3: (Auricular acupuncture):ti,ab,kw OR (Auricular):ti,ab,kw OR (Ear):ti,ab,kw OR (Acupressure):ti,ab,kw OR (Electro-acupuncture):ti,ab,kw OR (Stimulation):ti,ab,kw [**61632**]

#4: #1 AND #2 AND #3 [**34**]

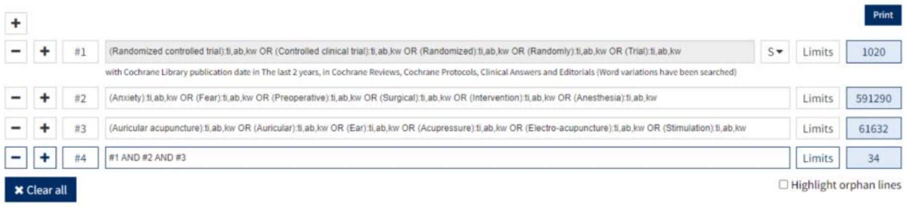

eMethods 5: Scopus Database Search Strategy Search strategy

Scopus Database (24,954)

#1: (TITLE-ABS-KEY (randomized AND controlled AND trial) OR TITLE-ABS-KEY (controlled AND clinical AND trial) OR TITLE-ABS-KEY (randomized) OR TITLE-ABS-KEY (randomly) OR TITLE-ABS-KEY (trial)) [**3,266,088**]

#2: (TITLE-ABS-KEY (anxiety) OR TITLE-ABS-KEY (fear) OR TITLE-ABS-KEY (preoperative) OR TITLE-ABS-KEY (surgical) OR TITLE-ABS-KEY (intervention) OR TITLE-ABS-KEY (anesthesia)) [**4,399,830**]

#3: (TITLE-ABS-KEY (auricular AND acupuncture) OR TITLE-ABS-KEY (auricular) OR TITLE-ABS-KEY (ear) OR TITLE-ABS-KEY (acupressure) OR TITLE-ABS-KEY (electro-acupuncture) OR TITLE-ABS-KEY (stimulation)) [**1,280,149**]

#4: #1 AND #2 AND #3 [**24,954**]

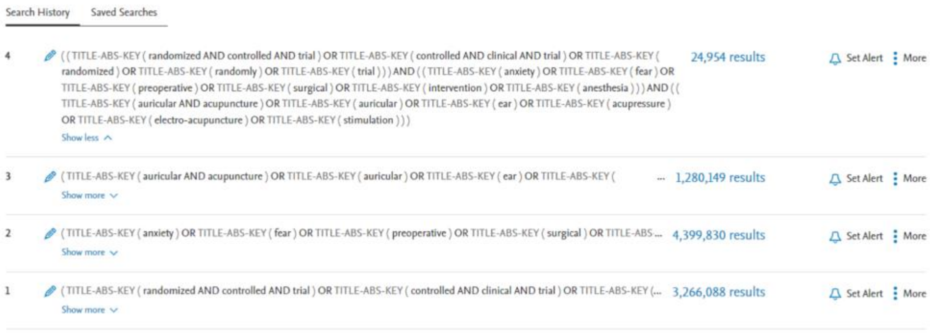

eTable 1: Baseline characteristics of included studies

First author, year	N of patients (female %)	Age (mean, years)	Surgery	Study intervention	Control intervention(s)	N of patients in		Type of anxiety assessment	Time to assess anxiety scores
						study group	control group(s)		
Gol, 2020	66 (84)	30	ENT	AuPrs	NI	33	33	STAI	After the Intervention
Karst, 2007	67 (45)	39	dental	AuPct	Sham/NI/Benz	19	48	STAI-S; STAI-T	30 minutes after intervention
Luo, 2016	43 (100)	36	gynecological	AuPrs	Sham	21	22	STAI-S; STAI-T	30 minutes after intervention
Michalek-Sauberer, 2012	182 (70)	38	dental	AuPct	Sham/NI	61	121	STAI-S; STAI-T	Before dental treatment
Qu, 2014	305 (100)	32	IVF	AuPrs	Sham/NI	101	204	STAI-S	The time-point on the morning of the

									day of embryo transfer
Wang, 2001	91 (73)	40	various	AuPct	Sham	31	59	STAI-S	30 minutes after intervention
Wang, 2007	56 (60)	45	lithotripsy	AuPct	Sham	29	27	STAI-S; STAI-T	30 minutes after intervention
Bang, 2020	24 (57)	66	cardiac surgery	AuPrs	Sham	21	21	STAI	Pre-op
Zanella, 2022	41 (34)	57	cholecystectomy or TEP	AuPct	NI/Benz	30	58	STAI	After treatment

Abbreviation: NR: not reported; ENT: ear nose throat surgery; TEP: totally extraperitoneal hernia repair; IVF: in vitro fertilization; AuPrs: auricular acupressure; AuES: electrical stimulation of the auricle; AuPct: auricular acupuncture; NI: no intervention; Benz: benzodiazepines; STAI: State Trait Anxiety Inventory; STAI-T: State Trait Anxiety Inventory for evaluating trait anxiety; STAI-S: State Trait Anxiety Inventory for state anxiety.

eTable 2: Risk of Bias Assessment

Domain	Signalling question	Response	Comments
Unique ID	1 — Study ID: Gol 2020	Assessor	Yexin
**Domain**	**Signalling question**	**Response**	**Comments**
Bias arising from the randomization process	1.1 Was the allocation sequence random?	Y	Patients were randomly divided into ear acupressure and control groups based on
	1.2 Was the allocation sequence concealed until participants were enrolled and assigned to interventions?	Y	
	1.3 Did baseline differences between intervention groups suggest a problem with the randomization process?	N	At baseline, there was no significant difference between groups.
	**Risk of bias judgement**	**Low**	
Bias due to deviations from intended interventions	2.1 Were participants aware of their assigned intervention during the trial?	N	None of the subjects were blind to the study.
	2.2 Were carers and people delivering the interventions aware of participants' assigned intervention during the trial?	PY	Carers and people delivering the
	2.3. If Y/PY/NI to 2.1 or 2.2: Were there deviations from the intended intervention that arose because of the experimental context?	N	Results were obtained by patient questionnaire, so participants were unlikely to interfere with the results.
	2.4 If Y/PY to 2.3: Were these deviations likely to have affected the outcome?	NA	
	2.5. If Y/PY/NI to 2.4: Were these deviations from intended intervention balanced between groups?	NA	
	2.6 Was an appropriate analysis used to estimate the effect of assignment to intervention?	Y	An appropriate analysis was performed.
	2.7 If N/PN/NI to 2.6: Was there potential for a substantial impact (on the result) of the failure to analyse participants in the	NA	
	**Risk of bias judgement**	**Low**	
Bias due to missing outcome data	3.1 Were data for this outcome available for all, or nearly all, participants randomized?	Y	The study started with 66 patients who were present until the end of the study.
	3.2 If N/PN/NI to 3.1: Is there evidence that the result was not biased by missing outcome data?	NA	
	3.3 If N/PN to 3.2: Could missingness in the outcome depend on its true value?	NA	
	3.4 If Y/PY/NI to 3.3: Is it likely that missingness in the outcome depended on its true value?	NA	
	**Risk of bias judgement**	**Low**	
Bias in measurement of the outcome	4.1 Was the method of measuring the outcome inappropriate?	N	The method of measuring the outcome was appropriate. The study started with 66 patients who were present until the end of the study.
	4.2 Could measurement or ascertainment of the outcome have differed between intervention groups?	N	No difference
	4.3 Were outcome assessors aware of the intervention received by study participants?	PY	The results were obtained by patient questionnaire
	4.4 If Y/PY/NI to 4.3: Could assessment of the outcome have been influenced by knowledge of intervention received?	N	Results were obtained by patient questionnaire, so participants were unlikely
	4.5 If Y/PY/NI to 4.4: Is it likely that assessment of the outcome was influenced by knowledge of intervention received?	NA	
	**Risk of bias judgement**	**Some concerns**	
Bias in selection of the reported result	5.1 Were the data that produced this result analysed in accordance with a pre-specified analysis plan that was finalized before unblinded outcome data were available for analysis?	N	This result was not mentioned in the registration protocol.
	5.2 ... multiple eligible outcome measurements (e.g. scales, definitions, time points) within the outcome domain?	N	
	5.3 ... multiple eligible analyses of the data?	N	
	**Risk of bias judgement**	**Some concerns**	
Overall bias	**Risk of bias judgement**	**Some concerns**	
Unique ID	2 — Study ID: Karst 2007	Assessor	Yexin
**Domain**	**Signalling question**	**Response**	**Comments**
Bias arising from the randomization process	1.1 Was the allocation sequence random?	Y	Before enrollment, patients were told that they would be randomly assigned to one
	1.2 Was the allocation sequence concealed until participants were enrolled and assigned to interventions?	PN	
	1.3 Did baseline differences between intervention groups suggest a problem with the randomization process?	N	At baseline, there was no significant difference between groups.
	**Risk of bias judgement**	**High**	
Bias due to deviations from intended interventions	2.1 Were participants aware of their assigned intervention during the trial?	PY	Patients were blinded regarding both acupuncture procedures. Blinding was not
	2.2 Were carers and people delivering the interventions aware of participants' assigned intervention during the trial?	PY	
	2.3. If Y/PY/NI to 2.1 or 2.2: Were there deviations from the intended intervention that arose because of the experimental context?	N	Before the study, no patient had experienced any of these techniques for reducing dental anxiety. Therefore, there was no specific patient expectation regarding any of the interventions.
	2.4 If Y/PY to 2.3: Were these deviations likely to have affected the outcome?	NA	
	2.5. If Y/PY/NI to 2.4: Were these deviations from intended intervention balanced between groups?	NA	
	2.6 Was an appropriate analysis used to estimate the effect of assignment to intervention?	Y	An appropriate analysis was performed.
	2.7 If N/PN/NI to 2.6: Was there potential for a substantial impact (on the result) of the failure to analyse participants in the	NA	
	**Risk of bias judgement**	**Low**	
Bias due to missing outcome data	3.1 Were data for this outcome available for all, or nearly all, participants randomized?	Y	Analyses included all subjects without missing data.
	3.2 If N/PN/NI to 3.1: Is there evidence that the result was not biased by missing outcome data?	NA	
	3.3 If N/PN to 3.2: Could missingness in the outcome depend on its true value?	NA	
	3.4 If Y/PY/NI to 3.3: Is it likely that missingness in the outcome depended on its true value?	NA	
	**Risk of bias judgement**	**Low**	
Bias in measurement of the outcome	4.1 Was the method of measuring the outcome inappropriate?	N	The method of measuring was appropriate
	4.2 Could measurement or ascertainment of the outcome have differed between intervention groups?	N	No difference
	4.3 Were outcome assessors aware of the intervention received by study participants?	Y	Blinding the acupuncture practitioner (BEF) was impossible because of methodological reasons.
	4.4 If Y/PY/NI to 4.3: Could assessment of the outcome have been influenced by knowledge of intervention received?	PN	Results were obtained by patient questionnaire, so participants were unlikely
	4.5 If Y/PY/NI to 4.4: Is it likely that assessment of the outcome was influenced by knowledge of intervention received?	NA	
	**Risk of bias judgement**	**Low**	
Bias in selection of the reported result	5.1 Were the data that produced this result analysed in accordance with a pre-specified analysis plan that was finalized	Y	Pre-specified outcomes reported.
	5.2 ... multiple eligible outcome measurements (e.g. scales, definitions, time points) within the outcome domain?	N	
	5.3 ... multiple eligible analyses of the data?	N	
	**Risk of bias judgement**	**Low**	
Overall bias	**Risk of bias judgement**	**High**	
Unique ID	3 — Study ID: Luo 2016	Assessor	Yexin
**Domain**	**Signalling question**	**Response**	**Comments**
Bias arising from the randomization process	1.1 Was the allocation sequence random?	Y	All subjects were randomized into 1) sham acupressure (SA) (control group) and 2) AA
	1.2 Was the allocation sequence concealed until participants were enrolled and assigned to interventions?	Y	
	1.3 Did baseline differences between intervention groups suggest a problem with the randomization process?	PN	At baseline, there was no significant difference between groups.
	**Risk of bias judgement**	**Low**	
Bias due to deviations from intended interventions	2.1 Were participants aware of their assigned intervention during the trial?	N	We performed a randomized, double-blind, controlled study to test whether AA has the
	2.2 Were carers and people delivering the interventions aware of participants' assigned intervention during the trial?	N	
	2.3. If Y/PY/NI to 2.1 or 2.2: Were there deviations from the intended intervention that arose because of the experimental	NA	
	2.4 If Y/PY to 2.3: Were these deviations likely to have affected the outcome?	NA	
	2.5. If Y/PY/NI to 2.4: Were these deviations from intended intervention balanced between groups?	NA	
	2.6 Was an appropriate analysis used to estimate the effect of assignment to intervention?	Y	An appropriate analysis was performed.

	2.7 If N/PN/NI to 2.6: Was there potential for a substantial impact (on the result) of the failure to analyse participants in the	NA	
	**Risk of bias judgement**	**Low**	
**Bias due to missing outcome data**	3.1 Were data for this outcome available for all, or nearly all, participants randomized?	Y	Analyses include all subjects without missing data.
	3.2 If N/PN/NI to 3.1: Is there evidence that result was not biased by missing outcome data?	NA	
	3.3 If N/PN to 3.2: Could missingness in the outcome depend on its true value?	NA	
	3.4 If Y/PY/NI to 3.3: Is it likely that missingness in the outcome depended on its true value?	NA	

Domain	Signalling question	Response	Comments
	**Risk of bias judgement**	**Low**	
**Bias in measurement of the outcome**	4.1 Was the method of measuring the outcome inappropriate?	N	The method of measuring was appropriate
	4.2 Could measurement or ascertainment of the outcome have differed between intervention groups?	N	No difference
	4.3 Were outcome assessors aware of the intervention received by study participants?	N	Data were recorded and analyzed by other two investigators respectively, who were blinded to group assignment
	4.4 If Y/PY/NI to 4.3: Could assessment of the outcome have been influenced by knowledge of intervention received?	NA	
	4.5 If Y/PY/NI to 4.4: Is it likely that assessment of the outcome was influenced by knowledge of intervention received?	NA	
	**Risk of bias judgement**	**Low**	
**Bias in selection of the reported result**	5.1 Were the data that produced this result analysed in accordance with a pre-specified analysis plan that was finalized	Y	Pre-specified outcomes reported.
	5.2 ... multiple eligible outcome measurements (e.g. scales, definitions, time points) within the outcome domain?	N	
	5.3 ... multiple eligible analyses of the data?	N	
	**Risk of bias judgement**	**Low**	
**Overall bias**	**Risk of bias judgement**	**Low**	
**Unique ID**	4 **Study ID** Michalek-sauberer 2012	**Assessor**	Ye xin
**Domain**	**Signalling question**	**Response**	**Comments**
**Bias arising from the randomization process**	1.1 Was the allocation sequence random?	Y	Using a computer generated randomization table provided by the Center for Medical
	1.2 Was the allocation sequence concealed until participants were enrolled and assigned to interventions?	Y	
	1.3 Did baseline differences between intervention groups suggest a problem with the randomization process?	N	Type of dental treatment varied significantly among the groups. However, this did not affect baseline anxiety.
	**Risk of bias judgement**	**Low**	
**Bias due to deviations from intended interventions**	2.1.Were participants aware of their assigned intervention during the trial?	Y	Participants were informed that the aim of this study was to determine which acupoint
	2.2.Were carers and people delivering the interventions aware of participants' assigned intervention during the trial?	Y	
	2.3. If Y/PY/NI to 2.1 or 2.2: Were there deviations from the intended intervention that arose because of the experimental context?	PN	Outcome data were assessed by an investigator (AG) who never had any patient contact
	2.4 If Y/PY to 2.3: Were these deviations likely to have affected the outcome?	NA	
	2.5. If Y/PY/NI to 2.4: Were these deviations from intended intervention balanced between groups?	NA	
	2.6 Was an appropriate analysis used to estimate the effect of assignment to intervention?	Y	An appropriate analysis was performed.
	2.7 If N/PN/NI to 2.6: Was there potential for a substantial impact (on the result) of the failure to analyse participants in the	NA	
	**Risk of bias judgement**	**Low**	
**Bias due to missing outcome data**	3.1 Were data for this outcome available for all, or nearly all, participants randomized?	Y	Analyses included all subjects without missing data.
	3.2 If N/PN/NI to 3.1: Is there evidence that result was not biased by missing outcome data?	NA	
	3.3 If N/PN to 3.2: Could missingness in the outcome depend on its true value?	NA	
	3.4 If Y/PY/NI to 3.3: Is it likely that missingness in the outcome depended on its true value?	NA	
	**Risk of bias judgement**	**Low**	
**Bias in measurement of the outcome**	4.1 Was the method of measuring the outcome inappropriate?	N	The method of measuring was appropriate.
	4.2 Could measurement or ascertainment of the outcome have differed between intervention groups?	N	No difference.
	4.3 Were outcome assessors aware of the intervention received by study participants?	Y	Participants were informed that the aim of this study was to determine which acupoint on the external ear is more effective in reducing preoperative anxiety.
	4.4 If Y/PY/NI to 4.3: Could assessment of the outcome have been influenced by knowledge of intervention received?	PY	While blinding in our study was adequate with regard to the acupuncture groups,
	4.5 If Y/PY/NI to 4.4: Is it likely that assessment of the outcome was influenced by knowledge of intervention received?	PY	
	**Risk of bias judgement**	**High**	
**Bias in selection of the reported result**	5.1 Were the data that produced this result analysed in accordance with a pre-specified analysis plan that was finalized	Y	Pre-specified outcomes reported.
	5.2 ... multiple eligible outcome measurements (e.g. scales, definitions, time points) within the outcome domain?	N	
	5.3 ... multiple eligible analyses of the data?	N	
	**Risk of bias judgement**	**Low**	
**Overall bias**	**Risk of bias judgement**	**High**	
**Unique ID**	5 **Study ID** Qu 2014	**Assessor**	Ye xin
**Domain**	**Signalling question**	**Response**	**Comments**
**Bias arising from the randomization process**	1.1 Was the allocation sequence random?	Y	Subjects were randomized into an AA group, a Sham-AA group or a control group with the
	1.2 Was the allocation sequence concealed until participants were enrolled and assigned to interventions?	Y	
	1.3 Did baseline differences between intervention groups suggest a problem with the randomization process?	N	At baseline, there was no significant difference between groups.
	**Risk of bias judgement**	**Low**	
**Bias due to deviations from intended interventions**	2.1.Were participants aware of their assigned intervention during the trial?	N	They were all blinded to the group assignment. The IVF clinicians and
	2.2.Were carers and people delivering the interventions aware of participants' assigned intervention during the trial?	N	
	2.3. If Y/PY/NI to 2.1 or 2.2: Were there deviations from the intended intervention that arose because of the experimental	NA	
	2.4 If Y/PY to 2.3: Were these deviations likely to have affected the outcome?	NA	
	2.5. If Y/PY/NI to 2.4: Were these deviations from intended intervention balanced between groups?	NA	
	2.6 Was an appropriate analysis used to estimate the effect of assignment to intervention?	Y	An appropriate analysis was performed.
	2.7 If N/PN/NI to 2.6: Was there potential for a substantial impact (on the result) of the failure to analyse participants in the	NA	
	**Risk of bias judgement**	**Low**	
**Bias due to missing outcome data**	3.1 Were data for this outcome available for all, or nearly all, participants randomized?	Y	Analyses included all subjects without missing data.
	3.2 If N/PN/NI to 3.1: Is there evidence that result was not biased by missing outcome data?	NA	
	3.3 If N/PN to 3.2: Could missingness in the outcome depend on its true value?	NA	
	3.4 If Y/PY/NI to 3.3: Is it likely that missingness in the outcome depended on its true value?	NA	
	**Risk of bias judgement**	**Low**	
**Bias in measurement of the outcome**	4.1 Was the method of measuring the outcome inappropriate?	N	The method of measuring was appropriate
	4.2 Could measurement or ascertainment of the outcome have differed between intervention groups?	N	No difference
	4.3 Were outcome assessors aware of the intervention received by study participants?	N	They were all blinded to the group assignment. The IVF clinicians and laboratory staff were blinded to the group assignment.
	4.4 If Y/PY/NI to 4.3: Could assessment of the outcome have been influenced by knowledge of	NA	

	intervention received?		
	4.5 If Y/PY/NI to 4.4: Is it likely that assessment of the outcome was influenced by knowledge of intervention received?	NA	
	**Risk of bias judgement**	**Low**	
**Bias in selection of the reported result**	5.1 Were the data that produced this result analysed in accordance with a pre-specified analysis plan that was finalized	Y	Pre-specified outcomes reported.
	5.2 ... multiple eligible outcome measurements (e.g. scales, definitions, time points) within the outcome domain?	N	
	5.3 ... multiple eligible analyses of the data?	N	
	**Risk of bias judgement**	**Low**	
**Overall bias**	**Risk of bias judgement**	**Low**	

**Unique ID**	6	**Study ID**	Wang 2001	**Assessor**	Ye xin

**Domain**	**Signalling question**	**Response**	**Comments**
**Bias arising from the randomization process**	1.1 Was the allocation sequence random?	PY	Participants were randomized to three intervention groups.
	1.2 Was the allocation sequence concealed until participants were enrolled and assigned to interventions?	N	
	1.3 Did baseline differences between intervention groups suggest a problem with the randomization process?	N	At baseline, there was no significant difference between groups.
	**Risk of bias judgement**	**High**	
**Bias due to deviations from intended**	2.1.Were participants aware of their assigned intervention during the trial?	PY	Blinding was not mentioned
	2.2.Were carers and people delivering the interventions aware of participants' assigned intervention during the trial?	PY	
	2.3. If Y/PY/NI to 2.1 or 2.2: Were there deviations from the intended intervention that arose because of the experimental	PY	Blinding was not mentioned
	2.4 If Y/PY to 2.3: Were these deviations likely to have affected the outcome?	PN	No subjects dropped out or switched groups
	2.5. If Y/PY/NI to 2.4: Were these deviations from intended intervention balanced between groups?	NA	

interventions	2.6 Was an appropriate analysis used to estimate the effect of assignment to intervention?	Y	An appropriate analysis was performed.
	2.7 If N/PN/NI to 2.6: Was there potential for a substantial impact (on the result) of the failure to analyse participants in the	NA	
	**Risk of bias judgement**	**Some concerns**	
**Bias due to missing outcome data**	3.1 Were data for this outcome available for all, or nearly all, participants randomized?	Y	Analyses included all subjects without missing data.
	3.2 If N/PN/NI to 3.1: Is there evidence that result was not biased by missing outcome data?	NA	
	3.3 If N/PN to 3.2: Could missingness in the outcome depend on its true value?	NA	
	3.4 If Y/PY/NI to 3.3: Is it likely that missingness in the outcome depended on its true value?	NA	
	**Risk of bias judgement**	**Low**	
**Bias in measurement of the outcome**	4.1 Was the method of measuring the outcome inappropriate?	N	The method of measuring was appropriate
	4.2 Could measurement or ascertainment of the outcome have differed between intervention groups?	N	No difference
	4.3 Were outcome assessors aware of the intervention received by study participants?	PY	Blinding was not mentioned
	4.4 If Y/PY/NI to 4.3: Could assessment of the outcome have been influenced by knowledge of intervention received?	PY	Results were obtained by patient questionnaire, so participants were unlikely
	4.5 If Y/PY/NI to 4.4: Is it likely that assessment of the outcome was influenced by knowledge of intervention received?	PN	
	**Risk of bias judgement**	**Some concerns**	
**Bias in selection of the reported result**	5.1 Were the data that produced this result analysed in accordance with a pre-specified analysis plan that was finalized	Y	Pre-specified outcomes reported.
	5.2 ... multiple eligible outcome measurements (e.g. scales, definitions, time points) within the outcome domain?	N	
	5.3 ... multiple eligible analyses of the data?	N	
	**Risk of bias judgement**	**Low**	
**Overall bias**	**Risk of bias judgement**	**High**	
**Unique ID**	7 **Study ID** Wang 2007	**Assessor**	Ye xin
**Domain**	**Signalling question**	**Response**	**Comments**
**Bias arising from the randomization process**	1.1 Was the allocation sequence random?	Y	The group assignment was generated by a computer-generated randomized number.
	1.2 Was the allocation sequence concealed until participants were enrolled and assigned to interventions?	Y	
	1.3 Did baseline differences between intervention groups suggest a problem with the randomization process?	N	At baseline, there was no significant difference between groups.
	**Risk of bias judgement**	**Low**	
**Bias due to deviations from intended interventions**	2.1.Were participants aware of their assigned intervention during the trial?	N	All patients were told that they might or might not experience vibration when the
	2.2.Were carers and people delivering the interventions aware of participants' assigned intervention during the trial?	N	
	2.3. If Y/PY/NI to 2.1 or 2.2: Were there deviations from the intended intervention that arose because of the experimental	NA	
	2.4 If Y/PY to 2.3: Were these deviations likely to have affected the outcome?	NA	
	2.5. If Y/PY/NI to 2.4: Were these deviations from intended intervention balanced between groups?	NA	
	2.6 Was an appropriate analysis used to estimate the effect of assignment to intervention?	Y	An appropriate analysis was performed.
	2.7 If N/PN/NI to 2.6: Was there potential for a substantial impact (on the result) of the failure to analyse participants in the	NA	
	**Risk of bias judgement**	**Low**	
**Bias due to missing outcome data**	3.1 Were data for this outcome available for all, or nearly all, participants randomized?	N	Two patients, However, did not complete the study because of logistical issues relating to the availability of study drugs
	3.2 If N/PN/NI to 3.1: Is there evidence that result was not biased by missing outcome data?	N	Not mentioned
	3.3 If N/PN to 3.2: Could missingness in the outcome depend on its true value?	N	However, did not complete the study because of logistical issues relating to the
	3.4 If Y/PY/NI to 3.3: Is it likely that missingness in the outcome depended on its true value?	NA	
	**Risk of bias judgement**	**Low**	
**Bias in measurement of the outcome**	4.1 Was the method of measuring the outcome inappropriate?	N	The method of measuring was appropriate
	4.2 Could measurement or ascertainment of the outcome have differed between intervention groups?	N	No difference
	4.3 Were outcome assessors aware of the intervention received by study participants?	N	Both ears were then covered with a surgical hat to conceal the needle locations and thus blind the observers.
	4.4 If Y/PY/NI to 4.3: Could assessment of the outcome have been influenced by knowledge of intervention received?	NA	
	4.5 If Y/PY/NI to 4.4: Is it likely that assessment of the outcome was influenced by knowledge of intervention received?	NA	
	**Risk of bias judgement**	**Low**	
**Bias in selection of the reported result**	5.1 Were the data that produced this result analysed in accordance with a pre-specified analysis plan that was finalized	Y	Pre-specified outcomes reported.
	5.2 ... multiple eligible outcome measurements (e.g. scales, definitions, time points) within the outcome domain?	N	
	5.3 ... multiple eligible analyses of the data?	N	
	**Risk of bias judgement**	**Low**	
**Overall bias**	**Risk of bias judgement**	**Low**	
**Unique ID**	8 **Study ID** Bang 2020	**Assessor**	Ye xin
**Domain**	**Signalling question**	**Response**	**Comments**
**Bias arising from the randomization process**	1.1 Was the allocation sequence random?	Y	Participants were randomly assigned to one of the two groups using
	1.2 Was the allocation sequence concealed until participants were enrolled and assigned to interventions?	PN	
	1.3 Did baseline differences between intervention groups suggest a problem with the randomization process?	N	At baseline, there was no significant difference between groups.
	**Risk of bias judgement**	**High**	
**Bias due to deviations from intended interventions**	2.1.Were participants aware of their assigned intervention during the trial?	N	To achieve a single-blind, a sham control was set up.
	2.2.Were carers and people delivering the interventions aware of participants' assigned intervention during the trial?	Y	
	2.3. If Y/PY/NI to 2.1 or 2.2: Were there deviations from the intended intervention that arose because of the experimental context?	N	Results were obtained by patient questionnaire, so participants were unlikely to interfere with the results
	2.4 If Y/PY to 2.3: Were these deviations likely to have affected the outcome?	NA	
	2.5. If Y/PY/NI to 2.4: Were these deviations from intended intervention balanced between groups?	NA	
	2.6 Was an appropriate analysis used to estimate the effect of assignment to intervention?	Y	An appropriate analysis was performed.
	2.7 If N/PN/NI to 2.6: Was there potential for a substantial impact (on the result) of the failure to analyse participants in the	NA	
	**Risk of bias judgement**	**Low**	
**Bias due to missing outcome data**	3.1 Were data for this outcome available for all, or nearly all, participants randomized?	Y	Participants (N = 42) were randomly assigned into either the experimental (n=21) or control group (n=21).
	3.2 If N/PN/NI to 3.1: Is there evidence that result was not biased by missing outcome data?	NA	
	3.3 If N/PN to 3.2: Could missingness in the outcome depend on its true value?	NA	
	3.4 If Y/PY/NI to 3.3: Is it likely that missingness in the outcome depended on its true value?	NA	
	**Risk of bias judgement**	**Low**	
	4.1 Was the method of measuring the outcome inappropriate?	N	The method of measuring was appropriate

**Bias in measurement of the outcome**	4.2 Could measurement or ascertainment of the outcome have differed between intervention groups?			N	No difference
	4.3 Were outcome assessors aware of the intervention received by study participants?			Y	A single-blind, randomized controlled trial
	4.4 If Y/PY/NI to 4.3: Could assessment of the outcome have been influenced by knowledge of intervention received?			N	Results were obtained by patient questionnaire, so participants were unlikely
	4.5 If Y/PY/NI to 4.4: Is it likely that assessment of the outcome was influenced by knowledge of intervention received?			NA	
	**Risk of bias judgement**			**Low**	
**Bias in selection of the reported result**	5.1 Were the data that produced this result analysed in accordance with a pre-specified analysis plan that was finalized			Y	Pre-specified outcomes reported.
	5.2 ... multiple eligible outcome measurements (e.g. scales, definitions, time points) within the outcome domain?			N	
	5.3 ... multiple eligible analyses of the data?			N	
	**Risk of bias judgement**			**Low**	
**Overall bias**	**Risk of bias judgement**			**High**	
**Unique ID**	9	**Study ID**	Zanella 2022	**Assessor**	Ye xin
**Domain**	**Signalling question**			**Response**	**Comments**
**Bias arising from the randomization process**	1.1 Was the allocation sequence random?			PY	Patients were randomized by means of closed envelope method to a control group.
	1.2 Was the allocation sequence concealed until participants were enrolled and assigned to interventions?			Y	

	1.3 Did baseline differences between intervention groups suggest a problem with the randomization process?	N	At baseline, there was no significant difference between groups.
	**Risk of bias judgement**	**Low**	
**Bias due to deviations from intended interventions**	2.1.Were participants aware of their assigned intervention during the trial?	PY	The Surgeons and anesthesiologists were blinded to the randomization of each patient.
	2.2.Were carers and people delivering the interventions aware of participants' assigned intervention during the trial?	N	
	2.3. If Y/PY/NI to 2.1 or 2.2: Were there deviations from the intended intervention that arose because of the experimental context?	PY	Not mentioned.
	2.4 If Y/PY to 2.3: Were these deviations likely to have affected the outcome?	PY	Possibly existing epistatic effects
	2.5. If Y/PY/NI to 2.4: Were these deviations from intended intervention balanced between groups?	PY	Not mentioned.
	2.6 Was an appropriate analysis used to estimate the effect of assignment to	Y	An appropriate analysis was performed.
	2.7 If N/PN/NI to 2.6: Was there potential for a substantial impact (on the result) of the failure to analyse participants in the group to which they were randomized?	NA	
	**Risk of bias judgement**	**Some concerns**	
**Bias due to missing outcome data**	3.1 Were data for this outcome available for all, or nearly all, participants randomized?	Y	Analyses included all subjects without missing data.
	3.2 If N/PN/NI to 3.1: Is there evidence that result was not biased by missing outcome data?	NA	
	3.3 If N/PN to 3.2: Could missingness in the outcome depend on its true value?	NA	
	3.4 If Y/PY/NI to 3.3: Is it likely that missingness in the outcome depended on its true value?	NA	
	**Risk of bias judgement**	**Low**	
**Bias in measurement of the outcome**	4.1 Was the method of measuring the outcome inappropriate?	N	The method of measuring was appropriate
	4.2 Could measurement or ascertainment of the outcome have differed between intervention groups?	N	No difference
	4.3 Were outcome assessors aware of the intervention received by study participants?	N	The Surgeons and anesthesiologists were blinded to the randomization of each patient.
	4.4 If Y/PY/NI to 4.3: Could assessment of the outcome have been influenced by knowledge of intervention received?	NA	
	4.5 If Y/PY/NI to 4.4: Is it likely that assessment of the outcome was influenced by knowledge of intervention received?	NA	
	**Risk of bias judgement**	**Low**	
**Bias in selection of the reported result**	5.1 Were the data that produced this result analysed in accordance with a pre-specified analysis plan that was finalized before unblinded outcome data were available for analysis?	Y	Pre-specified outcomes reported.
	5.2 ... multiple eligible outcome measurements (e.g. scales, definitions, time points) within the outcome domain?	N	
	5.3 ... multiple eligible analyses of the data?	N	
	**Risk of bias judgement**	**Low**	
**Overall bias**	**Risk of bias judgement**	**Low**	

eTable 3: Predefined subgroup analyses for primary outcome

**Subgroup**	**Subgroup category**	**Included studies**	**N of studies**	**WMD [95% CI]**	**Model**	***P***	***I^2* test (%)**
**Auricular stimulation compared with various control conditions**							
auricular acupressure	Sham group	Bang 2020, Luo 2016, Qu 2014	3	-6.38 [-12.04, -0.72]	Random	0.07	63
	no intervention	Gol 2020, Qu 2014	2	-10.01 [-17.29, -2.73]	Random	0.003	89
auricular acupuncture	Sham group	Karst 2007, Michalek-Sauberer 2012, Wang 2001, Wang 2007	4	-4.01 [-7.69, -0.33]	Random	0.05	62
	Midazolam group	Karst 2007, Zanella 2022	2	-0.69 [-2.90, 1.52]	Random	0.48	0
	no intervention	Karst 2007, Michalek-Sauberer 2012, Zanella 2022	3	-5.58 [-13.93, 2.77]	Random	＜0.00001	92

Abbreviation: N: Number; WMD: Weighted mean difference; CI: Confidence interval; I^2: I-square.

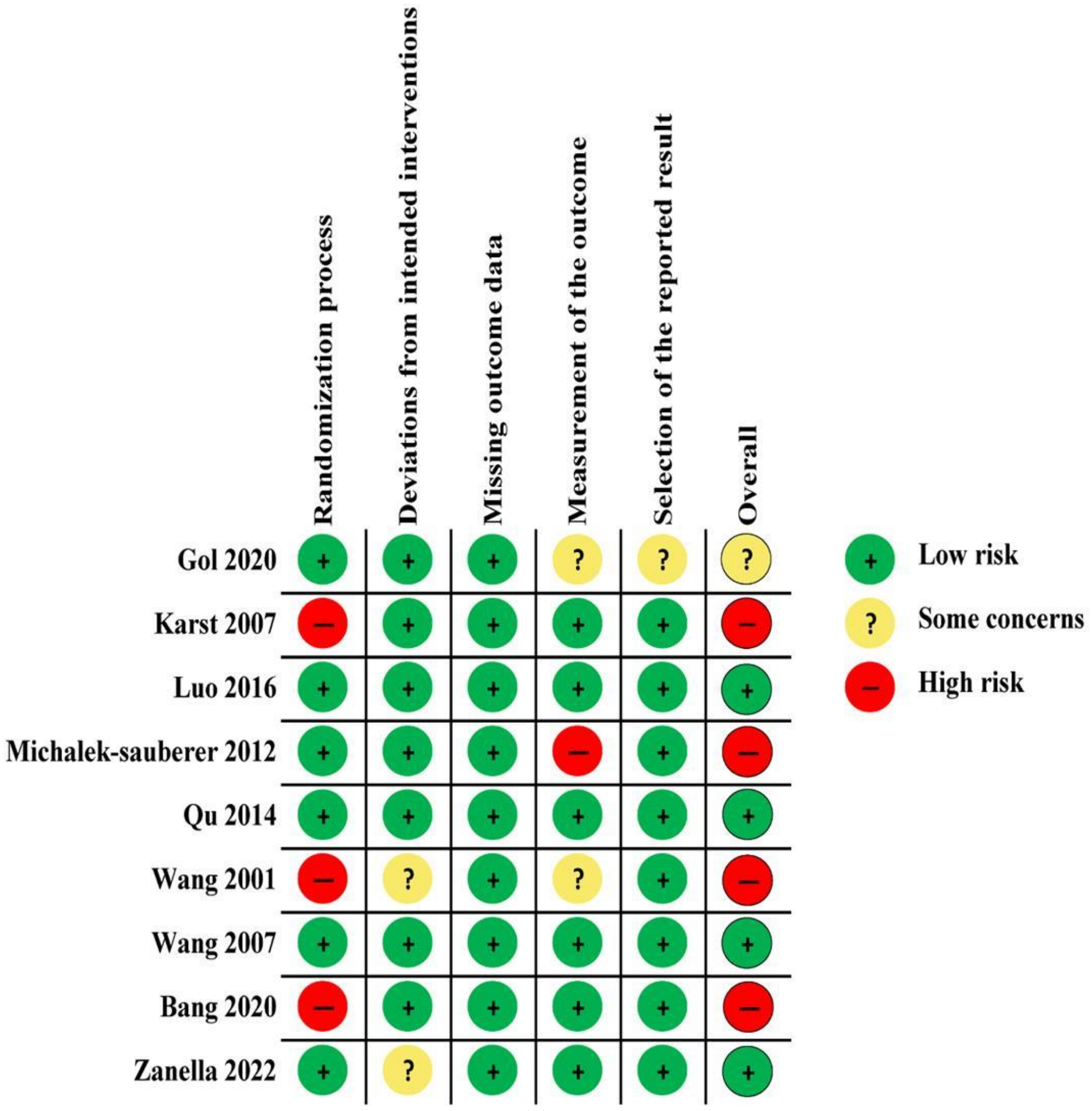

eFigure 1: Risk of bias summary

Review authors' judgements about the risk of bias of each domain in each study.
Green for low risk of bias, yellow for unclear risk of bias and red for high risk of bias.

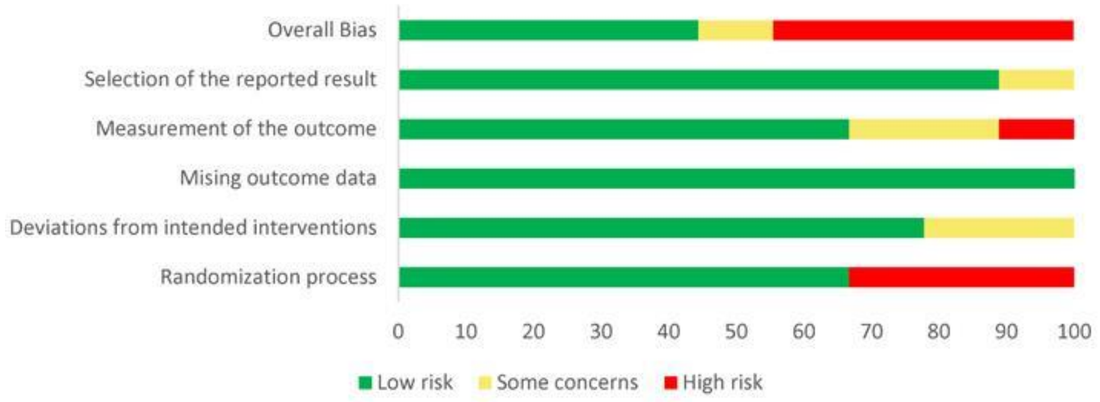

eFigure 2: Risk of bias graph

Review authors' judgements about each risk of bias item presented as percentages across all included studies. Green for low risk of bias, yellow for unclear risk of bias and red for high risk of bias.

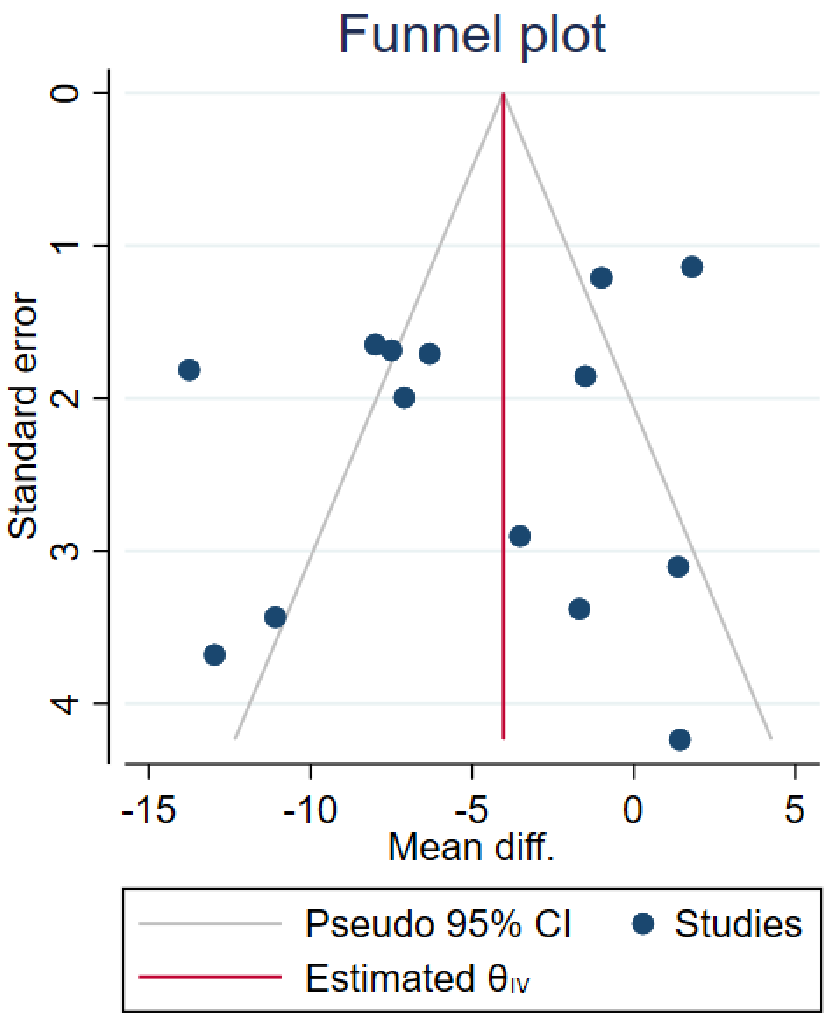

eFigure 3: Funnel Plot
